# The impact of circulatory arrest with selective antegrade cerebral perfusion on brain functional connectivity and postoperative cognitive function

**DOI:** 10.1038/s41598-023-40726-0

**Published:** 2023-08-23

**Authors:** Tae-Hoon Kim, Jooyoung Oh, Ha Lee, Myeong Su Kim, Seo-A. Sim, Sarang Min, Suk-Won Song, Jae-Jin Kim

**Affiliations:** 1 Department of Cardiovascular Surgery, Gangnam Severance Hospital, Yonsei University College of Medicine, Seoul, Republic of Korea; 2 Department of Psychiatry, Gangnam Severance Hospital, Yonsei University College of Medicine, Seoul, Republic of Korea; 3 Institute of Behavioral Sciences in Medicine, Yonsei University College of Medicine, Seoul, Republic of Korea; 4 Department of Cardiovascular Surgery, Ewha Womans University Aorta and Vascular Hospital, Seoul, Republic of Korea

**Keywords:** Attention, Consciousness, Aortic diseases

## Abstract

Aortic surgery is one of the most challenging types of surgeries, which is possibly related to cognitive sequelae. We aimed to investigate the changes in resting-state functional connectivity (rsFC) associated with intraoperative circulatory arrest (CA) in aortic surgery, exploring the relationship between the altered connectivity and postoperative cognitive functions. Thirty-eight patients participated in this study (14 with CA, 24 without). Functional magnetic resonance imaging was scanned on the fifth day after surgery or after the resolution of delirium if it was developed. We assessed the differences in the development of postoperative cognitive changes and rsFC between patients with and without CA. The occurrence of postoperative delirium and postoperative cognitive dysfunction was not significantly different between the patients with and without the application of CA. However, patients with CA showed increased in posterior cingulate cortex-based connectivity with the right superior temporal gyrus, right precuneus, and right hippocampus, and medial prefrontal cortex-based connectivity with the dorsolateral prefrontal cortex. The application of moderate hypothermic CA with unilateral antegrade cerebral perfusion is unlikely to affect aspects of postoperative cognitive changes, whereas it may lead to increased rsFC of the default mode network at a subclinical level following acute brain insults.

## Introduction

Postoperative delirium (POD) and postoperative cognitive dysfunction (POCD) frequently occur in elderly patients with major surgery, including aortic surgery^1–3^. POD represents acute brain failure and is characterized by an immediate onset of altered mental status with inattention, fluctuating cognition, and disorganized thinking^4^, whereas POCD includes subtle and diverse cognitive changes, including deficits in memory, intellectual performance, and executive functions^5^.

Although POD and POCD can be two manifestations of the same underlying spectrum^6^, the onset and course are distinctive^7^. POD symptoms usually peak after one to three days after surgery and disappears within few days^8,9^. On the other hand, POCD is more likely to appear one week after surgery, is observed up to several weeks after surgery, and can persist even for a very long time^10^.

Recently, the number of elderly adults undergoing aortic surgery is gradually increasing due to the aging of the population, advances in imaging modalities, and progress in surgical techniques for thoracic aortic aneurysm and dissection^11,12^. Considering this increase, the incidence of POD and POCD after aortic surgery is also expected to increase. Previously, both POD and POCD were once considered temporary and reversible phenomena rather than major complications after aortic surgery. However, recent studies have shown that they are not temporary and may result in irreversible cognitive dysfunction and/or long-term sequelae^13–15^. Furthermore, it is related to reduced quality of life and long-term morbidity and mortality^16–18^.

The potential pathophysiological mechanisms causing POD and POCD after cardiovascular surgery remain controversial; cerebral thromboembolism and ischemia, body temperature change, systemic inflammation, anesthesia itself, and blood–brain barrier dysfunction have been suggested^2,6,19^. In particular, in aortic arch surgery with hypothermic circulatory arrest (CA), cerebral ischemia and thromboembolic risk during the operation are higher and changes in body temperature and systemic inflammation are more prominent than in other operations. In addition, aortic surgery itself, regardless of hypothermic CA, is a significant risk factor for POD and POCD; after aortic surgery, the incidence of POD showed up to 39%^20^, whereas that of POCD is reported to be as high as 34%^21,22^).

Although many studies have been conducted regarding the hypothermic CA strategy and techniques with cerebral perfusion, in relation to ischemic/hemorrhagic stroke and major complications^23,24^, few studies investigated POD and POCD related to aortic arch surgery and hypothermic CA^25^. Furthermore, the neural mechanisms causing POD or POCD after hypothermic CA remain to be undetermined. The use of brain imaging techniques to elucidate changes in brain activity and connectivity will help understand the mechanisms of both POD and POCD, and the neural impact of hypothermic CA in patients who undergo aortic surgery.

Another important point in the study of complications after CA is the comparison between cerebral protection mechanisms, which are unilateral and bilateral antegrade cerebral perfusions (ACP)^21^. Currently, these two ACP methods are most commonly used ways for brain protection, but there is still no consensus on the superiority of these two methods^26^. Intuitively, in terms of neural protection, bilateral method seems to be the better way, however, it has also been reported that unilateral method has a lower risk of permanent neurological deficits^27^. This controversy may be due to the effects of different surgical techniques and clinical settings, therefore, it is difficult to draw a definite conclusion by only examining the outcome itself. In this context, it is also essential to examine the changes in neural mechanisms related to cognitive functions after aortic surgery with ACP.

In the study of the neural mechanism of POD and POCD, one of suitable targets may be the default-mode network (DMN) that is an ensemble of several brain regions, showing a higher level of activity at rest than during demanding cognitive tasks^28^. Given that altered DMN activity is associated with ineffective cognitive functions^29^, and a disruption of the anticorrelation between the DMN and task-positive networks is related to a delirious state^30^, the DMN may be a crucial functional network affecting the neural mechanism of POD or POCD. In fact, disruption of functional connectivity in the DMN was identified in the POD group of patients who underwent femur neck fracture surgery^31^. Nonetheless, the neural mechanism of POD and POCD has not been investigated on the samples with aortic surgery. Given that the impact of moderate hypothermic CA with unilateral ACP is crucial factor on the degree of brain oxygenation^32^, the relationship between the CA and POD/POCD with respect to the neural mechanisms needs to be evaluated in the analysis of resting-state functional magnetic resonance imaging (rsfMRI).

In order to elucidate the neural mechanism of POD and POCD after aortic surgery, this study aimed to examine the possible relationship between the alterations in resting-state functional connectivity and changes in cognitive functions. Considering the possible major effect of CA, we hypothesized that the neural mechanisms of POD/POCD would differ between different types of surgery. In other words, we assumed that patients with CA would show impaired functional connectivity in the DMN compared to patients without CA. Considering we applied only unilateral ACP method, we also tried to identify any asymmetrical brain response after surgery.

## Results

### Participant characteristics, surgery procedure, and outcome details

Patients who underwent surgery with intraoperative moderate hypothermic CA with unilateral ACP or did not were referred to as the CA group and NCA group, respectively. There were 14 patients in the CA group and 24 patients in the NCA group. Demographics and clinical characteristics of the two groups are presented in Table 1. We found no statistically significant differences between the two groups in age, sex, IQCODE score, and frequency of medical conditions, except hypertension. Before the scan, seven patients (three in the CA group and four in the NCA group) were treated with one or two antipsychotics among haloperidol, quetiapine, and risperidone, with minimal effective doses for each patient.

**Table 1:** Demographics, procedural details, and postoperative outcome in patients who underwent surgery with circulatory arrest or did not.

	Circulatory arrest (n = 14)	Non-circulatory arrest (n = 24)	*p*	Effect size (*d*)	95% CI
Age, years	76.1 ± 8.6	73.2 ± 6.7	0.254	0.39	[− 0.27, 1.05]
Sex, male/female	7/7	15/9	0.510	0.22	[− 0.42, 0.85]
IQCODE	3.1 ± 0.1	3.2 ± 0.2	0.297	0.36	[− 0.30, 1.02]
Hypertension	11 (78.6%)	5 (20.8%)	0.002	1.15	[0.42, 1.89]
Diabetes	2 (14.3%)	2 (8.3%)	0.977	0.01	[− 0.63, 0.65]
Coronary artery disease	2 (14.3%)	0 (0%)	0.250	0.38	[− 0.27, 1.03]
COPD	1 (7.1%)	0 (0%)	0.782	0.09	[− 0.55, 0.73]
CVA	0 (0%)	0 (0%)			
Surgery					
Total arch replacement	8 (57.1%)				
Partial arch replacement	6 (42.9%)				
Abdominal aorta replacement		21 (87.5%)			
Thoracoabdominal aorta replacement		3 (12.5%)			
Circulatory arrest time, min	52.0 [47.0–57.0]				
Aortic cross clamp time, min	113.0 [100.0–122.0]				
Cardiopulmonary bypass time, min	142.5 [134.0–163.0]				
Operation time, min	309.0 [282.0–327.0]	114.0 [90.5–183.5]	< 0.001	1.34	[0.62, 2.07]
Postoperative outcome					
Overall mortality	0 (0%)	1 (4.2%)			
In-hospital mortality	0 (0%)	0 (0%)			
Postoperative stroke	0 (0%)	0 (0%)			
Silent embolic stroke on MRI	11 (78.6%)	4 (16.7%)	0.001	1.26	[0.51, 2.01]
Silent microhemorrhage on MRI	2 (14.3%)	3 (12.5%)	1.000	0.00	[− 0.64, 0.64]
Hospital stay, day	17.0 [14.0–23.0]	12.0 [8.5–19.0]	0.071	0.61	[− 0.05, 1.27]
ICU stay, day	2.0 [1.0–3.0]	1.0 [1.0–2.5]	0.011	0.91	[0.20, 1.60]

Values expressed as mean ± standard deviation or median [interquartile range] for continuous data and number (percent) for categorical data.

CI, confidence interval, COPD, chronic obstructive pulmonary disease; CVA, cerebrovascular accident; IQCODE, Informant Questionnaire on Cognitive Decline in the Elderly; rsfMRI, resting-state functional magnetic resonance imaging; ICU, intensive care unit.

In the CA group, eight patients (57.1%) underwent total arch replacement, and six had partial arch replacement (42.9%). In the NCA group, 21 patients (87.5%) underwent AAA open repair, and three TAAA open repair (12.5%). In the CA group, the median hypothermic CA time was 52.0 min [47.0–57.0], and the total operation time was significantly longer than for the NCA group (309.0 min [282.0–327.0] vs. 114.0 min [90.5–183.5], *p < *0.001). In the CA group, all patients except one underwent moderate hypothermic CA with unilateral ACP. Bilateral ACP was performed in one patient since the left regional brain oxygen saturation decreased > 15% from the baseline value. In the CA group, ICU stay and hospital stay were longer than in the NCA group (2.0 days [1.0–3.0] vs. 1.0 [1.0–2.5], *p =   *0.011; and 17.0 days [14.0–23.0] vs. 12.0 [8.5–19.0], *p =   *0.071, respectively). There was no clinically diagnosed postoperative stroke in either group. However, silent microhemorrhages and multiple microembolic infarctions were incidentally observed on the T1 and rsfMRI scans, and the incidence of acute microembolic infarctions was higher in the CA than in the NCA group (11 [78.6%] vs. 4 [16.7%], *p =   *0.001).

### Incidence of POD and POCD

Among the 38 patients, 21 patients (55.3%) developed POD within five days after surgery, and 17 patients did not experience POD until 5 days after surgery. In terms of POCD, 25 patients had the MMSE score of 26 or less (65.8%), while other 13 patients showed the MMSE score of 27 or higher.

Postoperative cognitive status is summarized in Table 2. In the CA group, eight patients suffered from POD (57.1%), and in the NCA group, 13 patients (54.2%). In terms of POCD, 9 patients in CA group showed impaired cognition (64.3%), while 16 patients in NCA group showed POCD (66.7%). The group difference in both POD and POCD incidence was not statistically significant. We also found no statistically significant differences between the two groups in onset and recovery day of POD and MMSE scores.

**Table 2:** Postoperative cognitive status in patients who underwent surgery with circulatory arrest or did not.

	Circulatory arrest (n = 14)	Non-circulatory arrest (n = 24)	*p*	Effect size (*d*)	95% CI
Postoperative delirium			0.859	0.06	[− 0.58, 0.69]
Presence	8 (57.1%)	13 (54.2%)			
Absence	6 (42.9%)	11 (45.8%)			
Onset of delirium, postoperative day^a^	2.9 ± 1.6	2.7 ± 1.1	0.808		
Recovery from delirium, postoperative day^a^	9.6 ± 4.9	9.5 ± 5.0	0.977	0.08	[− 0.58, 0.74]
Postoperative cognitive dysfunction			0.881	0.05	[− 0.59, 0.68]
Presence (MMSE-DS ≤ 26)	9 (64.3%)	16 (66.7%)			
Absence (MMSE-DS ≥ 27)	5 (35.7%)	8 (33.3%)			
MMSE	23.4 ± 6.0	24.4 ± 5.0	0.662	0.15	[− 0.51, 0.81]

Values expressed as mean ± standard deviation for continuous data and number (percent) for categorical data*.* MMSE-DS, Mini-Mental State Examination.

^a^Only in patients with delirium.

### fMRI data quality

When we compare the patients between patients who experienced delirium with the patients without delirium, we found significant differences in maximum motions and mean motions between two groups [maximum motions (mm), post-recovery from delirium: 0.18 ± 0.19 versus patients without delirium: 0.05 ± 0.04, (*p =   *0.02, t = 2.51, effect size: 0.82, C.I: 0.15 to 1.49), mean motions (mm), post-recovery from delirium: 0.05 ± 0.06 versus patients without delirium: 0.03 ± 0.05, (*p =   *0.03, t = 2.34, effect size: 0.76, CI 0.10–1.42)]. However, number of invalid scans [post-recovery from delirium: 2.24 ± 4.32 versus patients without delirium: 0.88 ± 1.49, (*p =   *0.23, t = 1.20, effect size: 0.39, CI − 0.25 to 1.04)], maximum global signal changes (%) [post-recovery from delirium: 5.80 ± 5.40 versus patients without delirium: 4.64 ± 2.87, (*p =   *0.44, t = 0.78, effect size: 0.26, CI − 0.38 to 0.90)], and mean global signal changes (%) [post-recovery from delirium: 0.88 ± 0.19 versus patients without delirium: 0.83 ± 0.10, (*p =   *0.40, t = 0.86, effect size: 0.28, CI − 0.36 to 0.92)] were not different between two groups. In terms of the comparison between CA group versus NCA group, significant differences were found in both maximum and mean global signal changes between two groups, while other parameters did not show any significant differences [number of invalid scans, CA group: 2.93 ± 4.82 versus NCA group: 0.88 ± 1.88, (*p =   *0.08, t = 1.81, effect size: 0.61, CI − 0.06 to 1.28), maximum motions (mm), CA group: 0.16 ± 0.20 versus NCA group: 0.10 ± 0.12, (*p =   *0.27, t = 1.12, effect size: 0.61, CI − 0.29 to 1.04), mean motions (mm), CA group: 0.03 ± 0.05 versus NCA group: 0.03 ± 0.05, (*p =   *0.95, t = 0.07, effect size: 0.02, CI − 0.64 to 0.68), maximum global signal changes (%), CA group: 7.19 ± 6.10 versus NCA group: 4.17 ± 2.60, (*p =   *0.05, t = 2.07, effect size: 0.69, CI 0.02–1.37), mean global signal changes (%), CA group: 1.10 ± 0.57 versus NCA group: 0.74 ± 0.24, (*p =   *0.01, t = 2.59, effect size: 0.87, CI 0.18–1.56)].

### Functional connectivity associated with the PCC and mPFC

**Figure 1:**
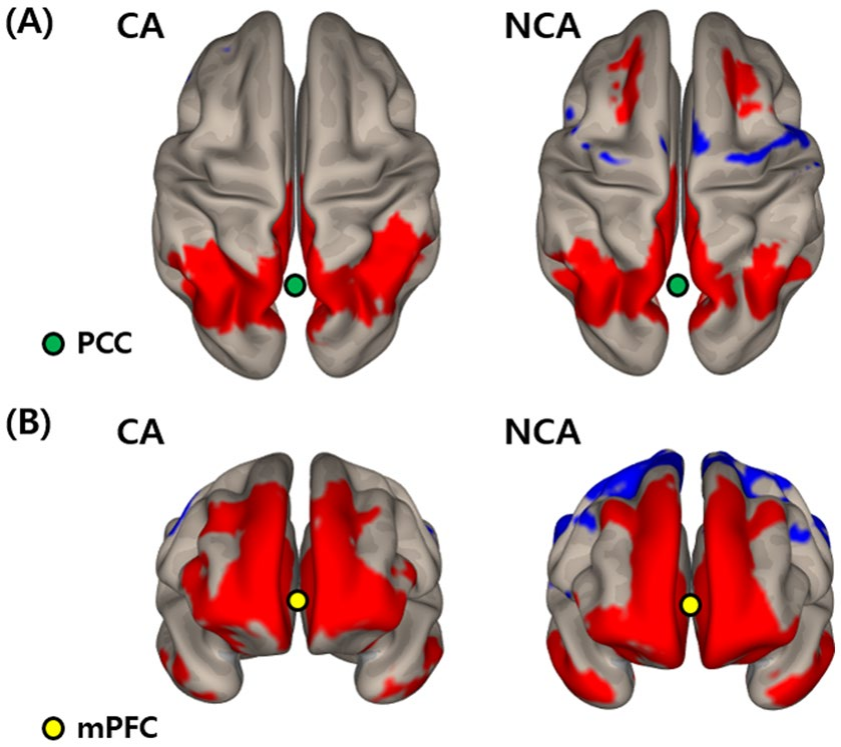
Functional connectivity with (**A**) the posterior cingulate cortex (PCC) and (**B**) medial prefrontal cortex (mPFC) in each group. Red color indicates positive correlation while blue color shows negative correlation. Multiple comparisons were corrected using a combination of voxel-level thresholds (significance considered for *p < *0.001) and cluster-extent threshold with false discovery rate correction (significance: *p < *0.05). Abbreviations: CA, circulatory arrest; NCA, non-circulatory arrest; PCC, posterior cingulate cortex; mPFC, medial prefrontal cortex.

**Figure 2:**
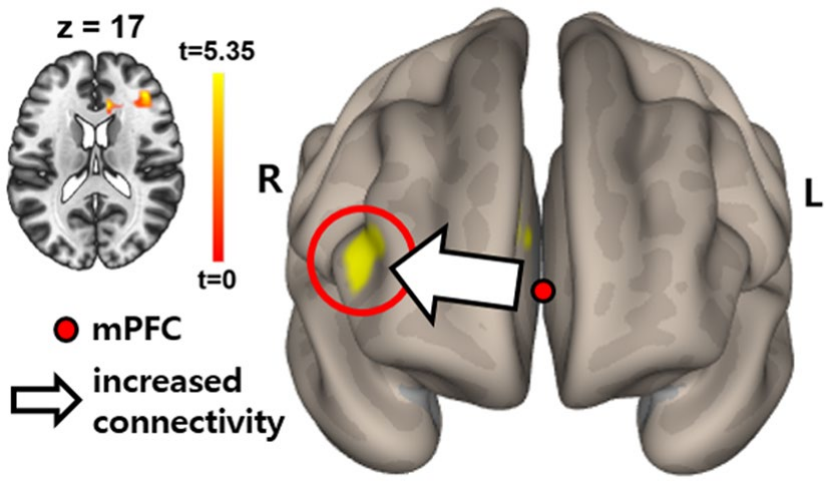
Increases connectivity between the medial prefrontal cortex and right dorsolateral prefrontal cortex (indicated by arrow and marked with yellow color) in circulatory arrest patients compared with non-circulatory arrest patients. Multiple comparisons were corrected using a combination of voxel-level thresholds (significance considered for *p < *0.001) and cluster-extent threshold with false discovery rate correction (significance: *p < *0.05). Abbreviations: R, right; L, left; mPFC, medial prefrontal cortex.

At a visual inspection of PCC-and mPFC-based connectivity, almost no anticorrelation was observed between the DMN and task-positive network regions, such as the dorsolateral prefrontal cortex (dlPFC) in the CA group, whereas it was clearly seen in the NCA group (Fig. 1). The statistical differences in PCC- and mPFC-based connectivity between the two groups are listed in Table 3. In the PCC-based connectivity, the CA group exhibited increased connections with the right superior temporal gyrus, precuneus, and hippocampus compared to the NCA group. In mPFC-based connectivity, the CA group showed increased functional connections with the dlPFC compared to the NCA group (Fig. 2).

**Table 3:** Brain regions exhibiting a significant difference in functional connectivity with the seed regions between groups.

Seed	Brain region	Size (mm^3^)	MNI coordinates (x, y, z)
PCC	CA > NCA		
	Right superior temporal gyrus	287	44, − 32, 16
	Right precuneus	219	16, − 72, 42
	Right hippocampus	141	28, − 42, 0
	CA < NCA		
	None		
mPFC	CA > NCA		
	Right dlPFC	571	38, 36, 18
	CA < NCA		
	None		

MNI, Montreal Neurological Institute; CA, circulatory arrest; NCA, non-circulatory arrest; PCC, posterior cingulate cortex, mPFC, medial prefrontal cortex; dlPFC, dorsolateral prefrontal cortex. All imaging analyses were corrected for multiple comparisons using a combination of voxel-level thresholds (significance considered for *p < *0.001) and cluster-extent threshold with false discovery rate correction (significance: *p < *0.05).

### Correlations between the cognitive scores and connectivity strengths

There was no significant correlation between the IQCODE scores or postoperative MMSE scores and the functional connectivity strengths in the brain regions that showed a significant difference between the CA and NCA groups.

## Discussion

In the present study, in order to elucidate the effects of CA on the postoperative cognitive and neural changes, we assessed the development of POD and POCD and resting-state functional connectivity after aortic surgery. There were a fairly large number of patients with aortic surgery as possible participants, but unfortunately, the number of patients actually used in the analysis was reduced to only 38 due to the strict application of exclusion criteria and the low consent rate for participation due to the characteristics of participants composed of critically ill elderly patients. Nonetheless, we were able to observe significant results on the influence of CA on fMRI findings after aortic surgery.

Contrary to our hypothesis, we could not find any difference in the incidence of both POD and POCD between the CA and NCA groups. Despite the burden of CA, which makes it difficult to provide sufficient cerebral oxygen during aortic surgery, this result might suggest the existence of a neural compensation mechanism. It should also be noted, however, that several cerebral protection strategies are employed during hypothermic CA to avoid negative outcomes, including major postoperative complications, severe stroke, and death, without a clear superiority of efficiency^33^. Cerebral protection strategies have been attempted with several techniques, such as deep hypothermic CA, moderate hypothermic arrest with ACP (unilateral/bilateral), and retrograde cerebral perfusion. Recently, moderate hypothermic CA with unilateral or bilateral ACP technique is preferred^34^. For physiologic and sufficient cerebral perfusion, the bilateral antegrade SCP would be ideal; however, it interferes with the surgical field exposure and can cause complications related to the insertion of the cerebral perfusion catheter (intimal injury or embolization). Therefore, many institutions prefer unilateral antegrade SCP since there is no significant difference in major clinical outcomes, and it is the most convenient method. As a result of supporting this trend, our findings suggest that moderate hypothermic CA with unilateral ACP is successfully applied to the brain protection strategies to the extent that it does not significantly affect the cognitive changes after aortic surgery. One thing to additionally note is that, in this study, every aortic surgery was performed by a surgeon with high annual case volume and high cumulative years of practice experience. In fact, small case volume (five or fewer case annually) and low experience (≤ 5 years) was associated with major complications including myocardial infarction, respiratory failure, renal failure and mortality^35^. Therefore, if a surgeon was inexperienced, CA may have affected the cognitive changes.

Although we could not find the difference in the development of both POD and POCD between the CA and NCA groups, we found the group difference in functional connectivity after surgery. We found that the CA group showed increased connectivity with the PCC and the right superior temporal gyrus, precuneus, and hippocampus. The right superior temporal gyrus was known to play a role in visual search and spatial processing^36^, which functions are essential to maintain attention and awareness. The precuneus also has a core role in cognition through simultaneous interactions with both the DMN and frontoparietal networks^37^, which suggests that it integrates both internally and externally driven information^38^. The hippocampus is also an important region for normal cognition and behavior^39^. Considering the close relationship of these regions with cognitive functions, abnormally increased connectivity of these areas with the PCC as a reactive change after CA could be related to subclinical cognitive changes, which may finally result in long-term cognitive complications. In fact, a prior animal study showed that abnormal activation of the hippocampal glial cells after surgery is a possible mechanism of POCD^40^.

We also found the increase in connectivity between the mPFC and DLPFC after surgery. This result means a reduced anticorrelation between the resting-state brain network and task-positive network, similar to the results from delirium patients in our previous investigations^30,31^; these findings suggest that a similar alteration occurred in our CA patients. It should be noted that the anticorrelated and competitive relationship between the resting-state brain network and the task-positive network impacts behavior and cognitive functions^41,42^, and the loss of anticorrelation between these two networks is associated with cognitive impairment^43^. In particular, this loss of anticorrelation can lead to the activation of local reactive increases in connectivity^44^, in line with our findings in both mPFC- and PCC-based connectivity in CA patients.

In this study, patients with moderate hypothermic CA with unilateral ACP demonstrated a functional connectivity change limited to the right hemisphere, compared with NCA patients. This finding may suggest that the moderate hypothermic CA with unilateral ACP might be insufficient to maintain adequate perfusion to the whole brain. The main differences between the CA and NCA groups were hypothermic CA and unilateral (right side) ACP during surgery. Only the unilateral ACP may explain the unilateral functional connectivity changes. Overall, we can conclude that moderate hypothermic CA and unilateral ACP are associated with changes in functional connectivity to the right hemisphere. Considering we supplied more blood flow to the right hemisphere during unilateral ACP, increased connectivity is thought of a reactive change after CA with ACP rather than a compensation mechanism. In line with this, the concept of luxury perfusion was previously introduced^45^. In hypothermic condition where large amount of blood is not required, relatively excessive blood flow could result in increased intracerebral pressures that ultimately cause endothelial damage and cerebral edema^46^. In other words, the blood flow that we thought was appropriate during the operation may have been excessive. It seems necessary to verify the meaning of the subclinical brain connectivity changes found in this study through a future study examining long-term cognitive outcomes and neuroimaging data.

There were several limitations in this study. First, a small number of patients were enrolled, and thus the number of patients in the subgroups was insufficient to draw a meaningful conclusion. The limitations mentioned can ultimately lead to issues in the reproducibility of the results, and therefore, careful interpretation should be exercised. In addition, only elderly patients (> 60 years) were enrolled in this study, and thus it remains to be determined whether the same results in terms of the postoperative cognitive and neural changes are identified in younger patients. Second, since we did not follow up with the patients for long-term results, it is unknown whether POCD after aortic surgery will be temporary or permanent. Furthermore, because baseline cognitive function was evaluated only indirectly by the IQCODE scores, preoperative cognitive states may not have been accurately reflected. In cases where the patient's condition permits, it would have been ideal to measure baseline MMSE before surgery to examine the possible correlation between changes in cognitive function and resting-state functional connectivity. Third, CA was conducted only with unilateral ACP, and thus it is not known that CA with bilateral ACP will produce the same phenomena. Further longitudinal research in patients with aortic root surgery without hypothermic CA, and aortic arch surgery with unilateral and bilateral antegrade SCP, is needed to confirm the impact of CA and cerebral protection strategies on postoperative cognitive function and brain functional connectivity.

It should be also discussed about the reliability issues^47^. In studies with a limited sample size, several factors such as interindividual differences and test–retest reliability can significantly influence the reliability of the data. It is essential to consider that our participants were either in a state without delirium or in a state after recovering from delirium. In addition, they also experienced either CA or NCA during surgery. Therefore, it is important to acknowledge that the tests were not conducted under the same conditions. However, our study has several strengths in terms of reliability. Firstly, we specifically investigated the DMN, which is known to have relatively fewer reliability issues. Secondly, we used a single scanner, ensuring consistency in data acquisition. Additionally, we made sure that all subjects maintained the same state of having their eyes closed, minimizing potential variability. Another advantage of our study is that we did not employ global signal regression, which is considered beneficial for reliability.

We found that moderate hypothermic CA with unilateral ACP did not influence on the incidence of both POD and POCD after aortic surgery, suggesting that the application of CA is unlikely to affect aspects of postoperative cognitive changes. In the neuroimaging results, however, patients with CA showed increased in PCC-based connectivity with the right superior temporal gyrus, right precuneus, and right hippocampus, and mPFC-based connectivity with the dlPFC. These findings suggest that CA may lead to increases in functional connectivity of the default mode network at a subclinical level following acute brain insults. Follow-up studies with large sample sizes are needed to determine whether this brain changes will remain at the subclinical level even after time has elapsed.

## Methods

### Participants

**Figure 3:**
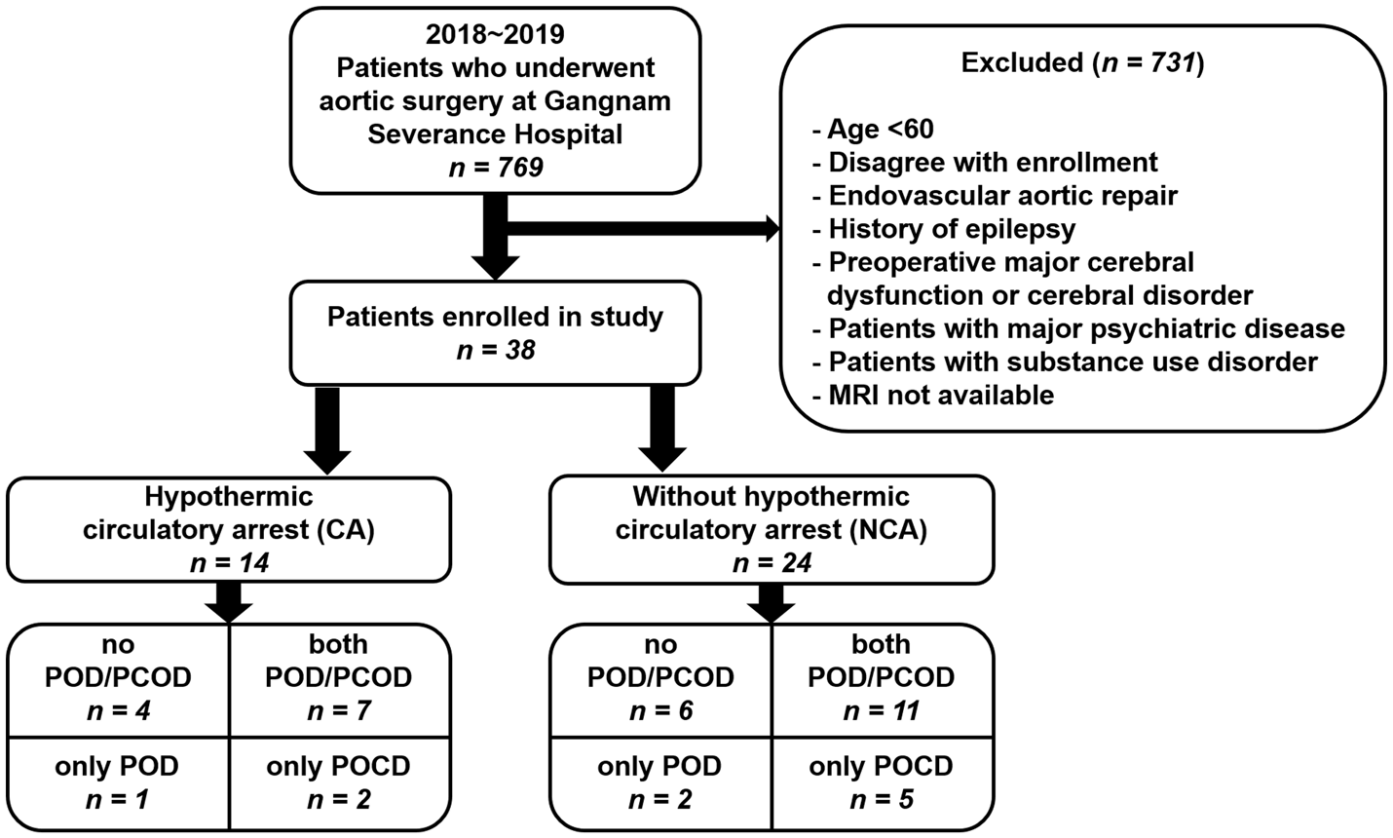
Study flowchart and POD/POCD outcomes of enrolled patients. Abbreviations: CA, circulatory arrest; NCA, non-circulatory arrest; POD, postoperative delirium; POCD, Postoperative cognitive dysfunction.

This study was performed on patients > 60 years of age who underwent aortic surgery with and without moderate hypothermic CA with ACP (including arch replacement, thoracoabdominal aortic aneurysm [TAAA], and abdominal aorta aneurysm [AAA]) at Yonsei University Gangnam Severance Hospital from 2018 to 2019. A total of 769 patients underwent aortic surgery during this period. After surgery, we explained the study to patients and their surrogates, and acquired informed consent. Exclusion criteria were under 60 years of age, disagree with enrollment, endovascular aortic repair, patients with epilepsy history, preoperative major cerebral dysfunction or cerebral disorder, major psychiatric diseases, or substance use disorders, and contraindication for MRI scanning (Fig. 3). Thirty-eight patients who consented to participate in the study and successfully underwent MRI were finally selected as study subjects. This study was approved by the institutional review board of Yonsei University Gangnam Severance Hospital.

### Aortic surgery (Cardiopulmonary bypass and circulatory arrest strategy)

The procedure began with a median sternotomy and standard cardiopulmonary bypass through side-graft cannulation of both right axillary and femoral artery. The Y connector was mounted on the arterial line, dividing the flow into two lines: one to the right axillary cannula and the other to the femoral cannula. Systemic cooling began shortly after the cardiopulmonary bypass was established. When the core temperature reached approximately 28 °C, the right brachiocephalic artery was clamped, and unilateral antegrade selective cerebral perfusion (SCP) was initiated. Bilateral antegrade SCP was performed only when the regional brain oxygen saturation decreased > 15% from the baseline value. Cerebral flow started at a rate of 10 mL/kg/min and was adjusted according to the right radial artery pressure (60 mmHg) (Song et al., 2013). All surgical procedures were performed by a single surgeon, and the surgical strategy (CA, cerebral protection and cardiopulmonary bypass) was consistent during the study period.

### Assessment of cognitive function and MRI scanning

We daily assessed the occurrence of POD by the Confusion Assessment Method (CAM) for general ward patients (Wei et al., 2008) or CAM for the intensive care unit (CAM-ICU) (Ely et al., 2001) for ICU patients. If delirium did not develop by five days after surgery, the patient underwent structural MRI and rsfMRI scans at the day. If a patient experienced delirium within 5 days after surgery, the patient underwent the MRI scans after delirium was resolved. The baseline cognitive function was assessed through the surrogates’ report using the Informant Questionnaire on Cognitive Decline in the Elderly (IQCODE)^48^. At the time of MRI acquisition, the Mini-Mental State Examination (MMSE)^49^ was conducted to evaluate the postoperative cognitive function. According to the previous report, we set the cutoff value of the MMSE for POCD as 26/27^50^.

### Image acquisition and preprocessing

Postoperative high-resolution T1-weighted imaging and rsfMRI scans were obtained with a Signa EXITE 3.0 Tesla MR system (GE Healthcare, Chicago, IL, USA). T1-weighted images were acquired using a spoiled gradient-echo sequence (matrix = 256 × 256, echo time = 3.2 ms, repetition time = 8.2 ms, field of view = 240 mm, slice thickness = 1.2 mm, flip angle = 12°, and number of slices = 136) to serve as an anatomical reference for brain activity. Functional images were scanned for five minutes using gradient-echo echo-planar imaging sequences (matrix = 64 × 64, echo time = 17.6 ms, repetition time = 2,000 ms, field of view = 240 mm, slice thickness = 3 mm, flip angle = 90°, and number of slices = 50). The participants were instructed to rest with their eyes closed during the scan. We discarded the first 10 s of time series data to eliminate any signal decay related to the magnetization reaching equilibrium. The preprocessing step for the functional data included slice-timing correction, head motion correction, co-registration to the T1-weighted image for each participant, spatial normalization to the Montreal Neurological Institute template, and smoothing with a 6-mm full-width at half-maximum Gaussian filter, using the standard pipeline provided in the functional connectivity toolbox (CONN) v.18a (www.nitrc.org/projects/conn) and Statistical Parametric Mapping (SPM) 12 software (www.fil.ion.ucl.ac.uk/spm/software/). The aCompCor method was used as implemented in the CONN toolbox to correct artifacts, including spiking and motion^51,52^. The white matter, cerebrospinal fluid, and realignment parameters were considered confounders in a first-level analysis, and a band-pass filter was applied from 0.008 to 0.09 Hz. This denoising step addressed the confounding effects of participant movement without regressing the global signal and without affecting the intrinsic functional connectivity^53^. The data quality of fMRI data was assessed using following parameters: number of invalid scans (with an outlier threshold for scan-to-scan motions at 0.5 mm), maximum motions (mm), mean motions (mm), maximum global signal change (%), mean global signal change (%).

### Regions of interest

As seed regions, we considered the posterior cingulate cortex (PCC) and medial prefrontal cortex (mPFC) in the DMN, key regions related to POD and POCD^30,31^. These two regions were defined as a 10-mm sphere centered at previously defined coordinates: the PCC at x/y/z = − 0/− 56/28 and the mPFC at 0/54/− 8^54,55^. We manually confirmed the location of the spheres for each participant was placed within the boundaries of the predefined regions.

### Statistical analysis and functional connectivity analysis

Independent t-tests or Mann–Whitney U tests were conducted to compare continuous variables such as demographics and clinical characteristics between groups and chi-square analyses for categorical variables. Based on the Shapiro–Wilk test, if normality was satisfied, a parametric test was used, otherwise a non-parametric test. For the functional connectivity analysis, a seed-based correlation approach was utilized. Using the CONN toolbox, seed-to-voxel functional connectivity maps were generated for each participant. Voxel-wise correlations were calculated between the time series of seed regions and the whole brain. Correlation coefficients were converted to z values using Fisher’s r-to-z transformation to estimate the functional connectivity strengths. One-sample t-tests were conducted to determine group-level significance, and two-sample t-tests to analyze the differences between groups. All imaging analyses were corrected for multiple comparisons using a combination of voxel-level thresholds (significance considered for *p < *0.001) and cluster-extent threshold with false discovery rate correction (significance: *p < *0.05). The correlations between regional functional connectivity strengths (beta values extracted from each participant’s results in the clusters showing a significant group difference) and baseline/postoperative cognitive function (IQCODE or MMSE score) were calculated using the correlation analysis tools implemented in the CONN toolbox.

### Ethical approval

All procedures performed in studies involving human participants were in accordance with the ethical standards of the institutional and/or national research committee and with the 1964 Declaration of Helsinki and its later amendments or comparable ethical standards. Written informed consent was obtained from all participants including the patients and their surrogates.

### Consent to participate

Informed consent was obtained from all individual participants included in the study.

## Data Availability

The datasets generated and analyzed during the current study are not publicly available due the fact that they constitute an excerpt of research in progress but are available from the corresponding author on reasonable request.
